# Comparative metabolomic analysis highlights the involvement of sugars and glycerol in melatonin-mediated innate immunity against bacterial pathogen in *Arabidopsis*

**DOI:** 10.1038/srep15815

**Published:** 2015-10-28

**Authors:** Yongqiang Qian, Dun-Xian Tan, Russel J. Reiter, Haitao Shi

**Affiliations:** 1 Hainan Key Laboratory for Sustainable Utilization of Tropical Bioresources, College of Agriculture, Hainan University, Haikou, 570228, China; 2 State Key Laboratory of Tree Genetics and Breeding, Research Institute of Forestry, Chinese Academy of Forestry, Beijing, 100091, China; 3 Department of Cellular and Structural Biology, The University of Texas Health Science Center, San Antonio, TX, USA

## Abstract

Melatonin is an important secondary messenger in plant innate immunity against the bacterial pathogen *Pseudomonas syringe pv. tomato* (*Pst*) DC3000 in the salicylic acid (SA)- and nitric oxide (NO)-dependent pathway. However, the metabolic homeostasis in melatonin-mediated innate immunity is unknown. In this study, comparative metabolomic analysis found that the endogenous levels of both soluble sugars (fructose, glucose, melibose, sucrose, maltose, galatose, tagatofuranose and turanose) and glycerol were commonly increased after both melatonin treatment and *Pst* DC3000 infection in *Arabidopsis*. Further studies showed that exogenous pre-treatment with fructose, glucose, sucrose, or glycerol increased innate immunity against *Pst* DC3000 infection in wild type (Col-0) *Arabidopsis* plants, but largely alleviated their effects on the innate immunity in SA-deficient NahG plants and NO-deficient mutants. This indicated that SA and NO are also essential for sugars and glycerol-mediated disease resistance. Moreover, exogenous fructose, glucose, sucrose and glycerol pre-treatments remarkably increased endogenous NO level, but had no significant effect on the endogenous melatonin level. Taken together, this study highlights the involvement of sugars and glycerol in melatonin-mediated innate immunity against bacterial pathogen in SA and NO-dependent pathway in *Arabidopsis*.

Since melatonin (N-acetyl-5-methoxytryptamine) was first discovered and examined in plants by Dubbels *et al.* and Hattori *et al.* in 1995^1^,^2^, it has drawn progressively more attention of plant scientists^3^,^4^,^5^,^6^,^7^,^8^,^9^,^10^,^11^,^12^,^13^,^14^,^15^,^16^,^17^,^18^,^19^,^20^,^21^,^22^,^23^,^24^,^25^,^26^,^27^,^28^,^29^,^30^,^31^,^32^,^33^,^34^. Initially, the studies focused on the distribution of melatonin in plants, by comparing the differences of endogenous melatonin levels in different plant organs and in numerous plant species, and investigating the effects of various stress treatments, senescence, light, dark, the location of growth, growth state, harvest time of seeds on the endogenous melatonin levels^3^,^4^,^5^,^6^,^7^,^8^,^9^,^10^,^11^,^12^,^13^,^14^. In recent ten years, plant biologists have paid more attentions to investigating the biological roles of melatonin in plants due to exogenous application of melatonin^15^,^16^,^17^,^18^,^19^,^20^,^21^,^22^,^23^,^24^,^25^,^26^,^27^ or using melatonin-deficient and melatonin-enrich transgenic plants by genetic modulation of melatonin synthetic or metabolic-related genes^28^,^29^,^30^,^31^,^32^,^33^,^34^.

To date, the *in vivo* roles of melatonin in plant seed germination, primary root and lateral root development, circadian rhythms and photoprotection, flowering time and vegetative growth, fruit ripening and natural senescence have been described^15^,^16^,^17^,^18^,^19^,^20^. Also, melatonin protects against stresses including drought, salt, osmotic stress, oxidative stress, heat and cold stresses, copper stress, plant-pathogen interaction, etc^21^,^22^,^23^,^24^,^25^,^26^,^27^,^28^,^29^,^30^,^31^,^32^,^33^,^34^,^35^,^36^,^37^,^38^,^39^,^40^,^41^,^42^. Some biological roles of melatonin in plants are similar to those in animals, such as regulation of circadian rhythms^12^,^36^,^37^,^38^,^39^,^40^, scavenging of reactive nitrogen species (RNS) and reactive oxygen species (ROS)^13^,^42^,^43^,^44^,^45^,^46^, and regulation of innate immunity^22^,^32^,^33^,^41^,^47^,^48^.

In animals, melatonin is involved in innate immunity by acting on basophils, eosinophils, eosinophils, monocytes-macrophages, neutrophils, dendritic cells, mast cells and natural killer cells^47^,^48^. In plants, at least four reports have revealed the protective role of melatonin in plant innate immunity^22^,^32^,^33^,^41^. It was found that exogenous melatonin pre-treatment improved resistance of apple to Marssonina apple blotch (*Diplocarpon mali*) by modulating activities of antioxidant enzymes, hydrogen peroxide (H_2_O_2_) level and transcripts of pathogensis-related proteins (PRs) during plant-pathogen interaction^22^. Lee *et al.* found that melatonin-treatment increased disease resistance against pathogen attack such as Pseudomonas syringe pv. tomato (*Pst*) DC3000 by up-regulating a series of defense genes that were activated by salicylic acid (SA) and ethylene (ET) in *Arabidopsis* and tobacco^32^. Using the knockout mutant of serotonin N-acetyltransferase (SNAT) which is the penultimate enzyme in melatonin biosynthesis pathway, Lee *et al.* found that *snat* knockout mutants exhibited susceptibility to pathogen *Pst* DC3000 (*avrRpt2*) infection that coincided with decreased endogenous melatonin and SA levels as well as reduced induction of defense genes, this indicated that melatonin-elicited pathogen resistance is SA-dependent in *Arabidopsis*^33^. Recently, Shi *et al.* found that melatonin positively regulated plant innate immunity against bacterial pathogen in SA- and nitric oxide (NO)-dependent pathway^41^. Zhao *et al.* investigated the effect of exogenous melatonin on carbohydrate metabolism especially sucrose metabolism, and found that melatonin-mediated sucrose metabolism is contributed to pathogen resistance^49^. However, the comprehensive metabolic homeostasis including amino acids, organic acids, sugars and sugar alcohols in response to melatonin and bacterial pathogen remains unclear.

In this study, comparative metabolomic analysis using gas chromatography time-of-flight mass spectrometry (GC-TOF-MS) was performed to assay primary metabolites after melatonin treatment and *Pst* DC3000 infection in *Arabidopsis*. Further studies investigated the association among sugars and sugar alcohols, melatonin-mediated disease response, and SA and NO signaling in *Arabidopsis*. Based on collective results herein, we highlight the involvement of sugars and glycerol in melatonin-mediated innate immunity against bacterial pathogen in SA and NO-dependent pathway in *Arabidopsis*.

## Results

### The effects of exogenous melatonin treatment and *Pst* DC3000 infection on the endogenous levels of primary metabolites

**Figure 1:**
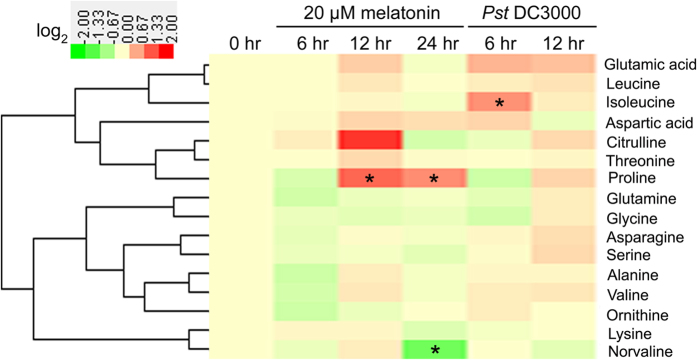
Heatmap showing the effects of exogenous melatonin treatment and *Pst* DC3000 infection on the endogenous levels of 16 amino acids. For exogenous melatonin treatments, 28-day-old of soil-grown WT (Col-0) *Arabidopsis* plants were watered with nutrient solution containing 20 μM melatonin from below in pots with plants. For pathogen infection, 28-day-old soil-grown WT (Col-0) *Arabidopsis* plant leaves were infected with bacterial suspension (OD_600_ = 0.002) containg 10 mM MgCl_2_ and 0.05% silwet L-77. For cluster analysis, all these metabolite levels were quantified as log_2_ value of fold change in relative to the WT plants at 0 hr of treatment which was set as 1.0. The detailed concentrations were shown in Table S1. The asterisk (*) indicates significant difference in comparison to 0 hr of treatment at *p *< 0.05.

**Figure 2:**
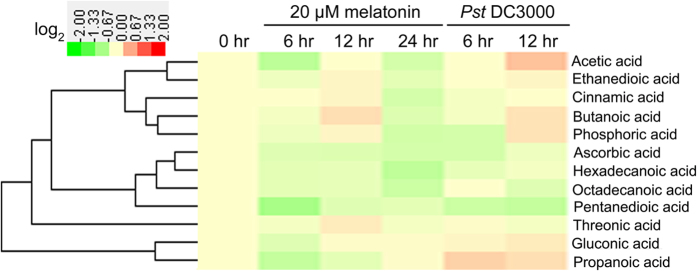
Heatmap showing the effects of exogenous melatonin treatment and *Pst* DC3000 infection on the endogenous levels of 12 organic acids. For exogenous melatonin treatments, 28-day-old of soil-grown WT (Col-0) *Arabidopsis* plants were watered with nutrient solution containing 20 μM melatonin from below in pots with plants. For pathogen infection, 28-day-old soil-grown WT (Col-0) *Arabidopsis* plant leaves were infected with bacterial suspension (OD_600_ = 0.002) containg 10 mM MgCl_2_ and 0.05% silwet L-77. For cluster analysis, all these metabolite levels were quantified as log_2_ value of fold change in relative to the WT plants at 0 hr of treatment which was set as 1.0. The detailed concentrations were shown in Table S1.

To gain additional insight into the metabolic homeostasis which is commonly affected by exogenous melatonin and bacterial pathogen *Pst* DC3000 infection, comparative metabolomic analysis using GC-TOF-MS was performed to quantify the primary metabolites after stress treatments. Totally, 51 metabolites including 16 amino acids, 12 organic acids, 18 sugars and 5 sugar alcohols were reproducibly examined in *Arabidopsis* leaves after different treatments (Table 1 and Supplemental Table S1). Among 16 assayed amino acids, exogenous melatonin significantly increased proline level after treatment for 12 hrs and 24 hrs, but reduced norvaline level after treatment for 24 hrs, and *Pst* DC3000 significantly increased isoleucine level after infection for 6 hrs (Fig. 1 and Supplemental Table 1). However, both exogenous melatonin treatment and *Pst* DC3000 infection had no significant effect on the endogenous levels of assayed 12 organic acids (Fig. 2 and Supplemental Table 1).

**Figure 3:**
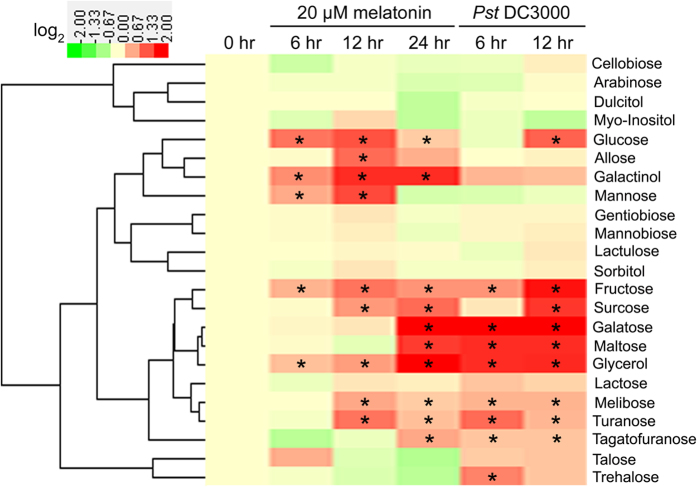
Heatmap showing the effects of exogenous melatonin treatment and *Pst* DC3000 infection on the endogenous levels of 18 sugars and 5 sugar alcohols. For exogenous melatonin treatments, 28-day-old of soil-grown WT (Col-0) *Arabidopsis* plants were watered with nutrient solution containing 20 μM melatonin from below in pots with plants. For pathogen infection, 28-day-old soil-grown WT (Col-0) *Arabidopsis* plant leaves were infected with bacterial suspension (OD_600_ = 0.002) containg 10 mM MgCl_2_ and 0.05% silwet L-77. For cluster analysis, all these metabolite levels were quantified as log_2_ value of fold change in relative to the WT plants at 0 hr of treatment which was set as 1.0. The detailed concentrations were shown in Table 1. The asterisk (*) indicates significant difference in comparison to 0 hr of treatment at *p *< 0.05.

Interestingly, both exogenous melatonin treatment and *Pst* DC3000 infection significantly increased the endogenous levels of multiple sugars and sugar alcohols (Fig. 3 and Table 1). Among these sugars and sugar alcohols, the endogenous levels of fructose, glucose, melibose, sucrose, maltose, galatose, tagatofuranose, turanose and glycerol commonly increased after melatonin treatment and *Pst* DC3000 infection (Fig. 3 and Table 1). The results indicate the possible involvement of sugars and sugar alcohols in melatonin-mediated plant disease response against bacterial pathogen *Pst* DC3000.

### The effects of exogenous sugar and glycerol treatments on plant disease resistance

**Figure 4:**
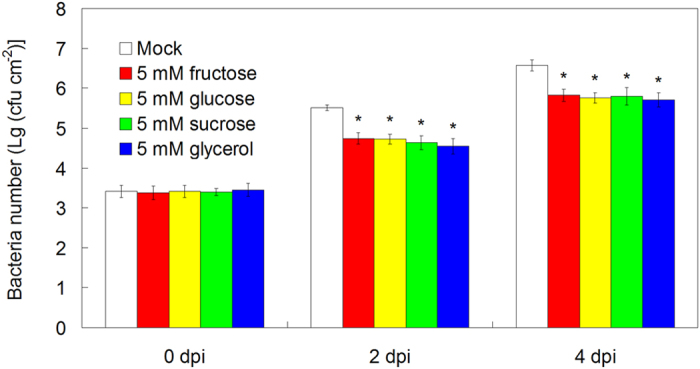
The effects of exogenous treatments of fructose, glucose, sucrose and glycerol on innate immunity against *Pst* DC3000 in *Arabidopsis*. For the assay, 26-day-old soil-grown WT (Col-0) *Arabidopsis* plants were watered with nutrient solution containing mock, 5 mM fructose, 5 mM glucose, 5 mM sucrose and 5 mM glycerol from below in pots with plants for 2 days, and then 28-day-old soil-grown WT (Col-0) *Arabidopsis* plant leaves were infected with bacterial suspension (OD_600_ = 0.002) containg 10 mM MgCl_2_ and 0.05% silwet L-77. At 0, 2 and 4 days post infection (dpi), the bacterial populations in the leave discs were determined. The results shown are the means ± SDs (n = 6), and *p* < 0.05 was considered as significant difference that was marked with asterisk (*).

To further investigate the possible relation among sugars and sugar alcohols, melatonin and plant disease resistance against bacterial pathogen *Pst* DC3000, we chose three popular sugars (fructose, glucose and sucrose) and glycerol for further analysis. Using quantification of bacteria number in *Pst* DC3000-infected leaves, we found that 5 mM fructose, or 5 mM glucose, or 5 mM sucrose, or 5 mM glycerol pre-treated plants showed significantly lower bacterial propagation at both 2 days post infection (dpi) and 4 dpi of *Pst* DC3000 (Fig. 4).

### Sugar and glycerol regulate plant disease resistance in SA- and NO-dependent pathway

**Figure 5:**
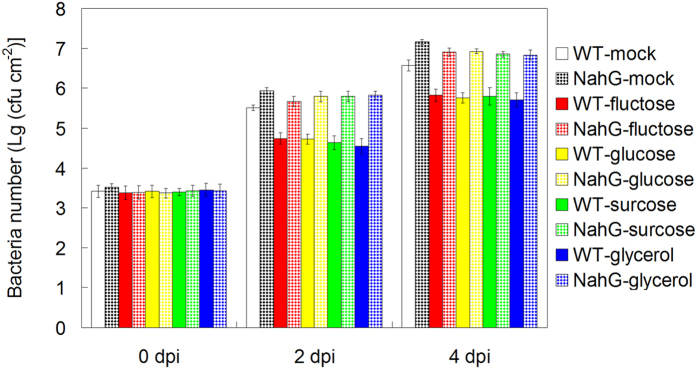
The involvement of SA in fructose, glucose, sucrose and glycerol-mediated innate immunity against *Pst* DC3000 in *Arabidopsis*. For the assay, 26-day-old soil-grown WT (Col-0) and NahG *Arabidopsis* plants were watered with nutrient solution containing mock, 5 mM fructose, 5 mM glucose, 5 mM sucrose and 5 mM glycerol from below in pots with plants for 2 days, and then 28-day-old soil-grown WT (Col-0) and NahG *Arabidopsis* plant leaves were infected with bacterial suspension (OD_600_ = 0.002) containg 10 mM MgCl_2_ and 0.05% silwet L-77. At 0, 2 and 4 days post infection (dpi), the bacterial populations in the leave discs were determined. The results shown are the means ± SDs (n = 6).

**Figure 6:**
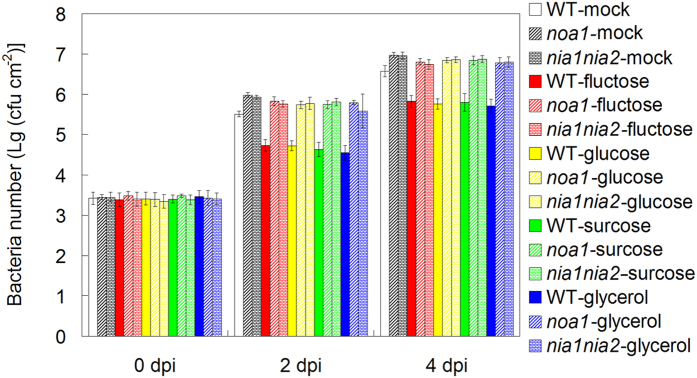
The involvement of NO in fructose, glucose, sucrose and glycerol-mediated innate immunity against *Pst* DC3000 in *Arabidopsis*. For the assay, 26-day-old soil-grown WT (Col-0) and NO deficient mutants (*noa1* and *nia1nia2*) were watered with nutrient solution containing mock, 5 mM fructose, 5 mM glucose, 5 mM sucrose and 5 mM glycerol from below in pots with plants for 2 days, and then 28-day-old soil-grown WT (Col-0) and NO deficient mutants (*noa1* and *nia1nia2*) were infected with bacterial suspension (OD_600_ = 0.002) containg 10 mM MgCl_2_ and 0.05% silwet L-77. At 0, 2 and 4 days post infection (dpi), the bacterial populations in the leave discs were determined. The results shown are the means ± SDs (n = 6).

Both SA and NO positively regulated innate immunity against *Pst* DC3000 infection, and they are essential for melatonin-mediated plant disease response in *Arabidopsis*^32^,^33^,^41^. To dissect how sugars (fructose, glucose and sucrose) and glycerol mediated disease resistance, we examined the effects of these sugars and sugar alcohols on wild type (WT, Col-0) plants, SA-deficient plants (NahG overexpressing plants) and NO deficient mutants (*noa1* and *nia1nia2*). Without sugars (fructose, glucose and sucrose) and glycerol pre-treatment, NahG, *noa1* and *nia1nia2* displayed significantly more bacterial propagation in comparison WT (Col-0) plants at both 2 dpi and 4 dpi of *Pst* DC3000 (Figs 5 and 6). Notably, the effect of 5 mM fructose, or 5 mM glucose, or 5 mM sucrose, or 5 mM glycerol pre-treatment on the disease resistance was alleviated in NahG, *noa1* and *nia1nia2* mutants, as evidenced by the bacterial propagation in *Pst* DC3000 infected leaves of these mutants (Figs 5 and 6).

### The effects of exogenous sugar and glycerol treatments on endogenous NO production

**Figure 7:**
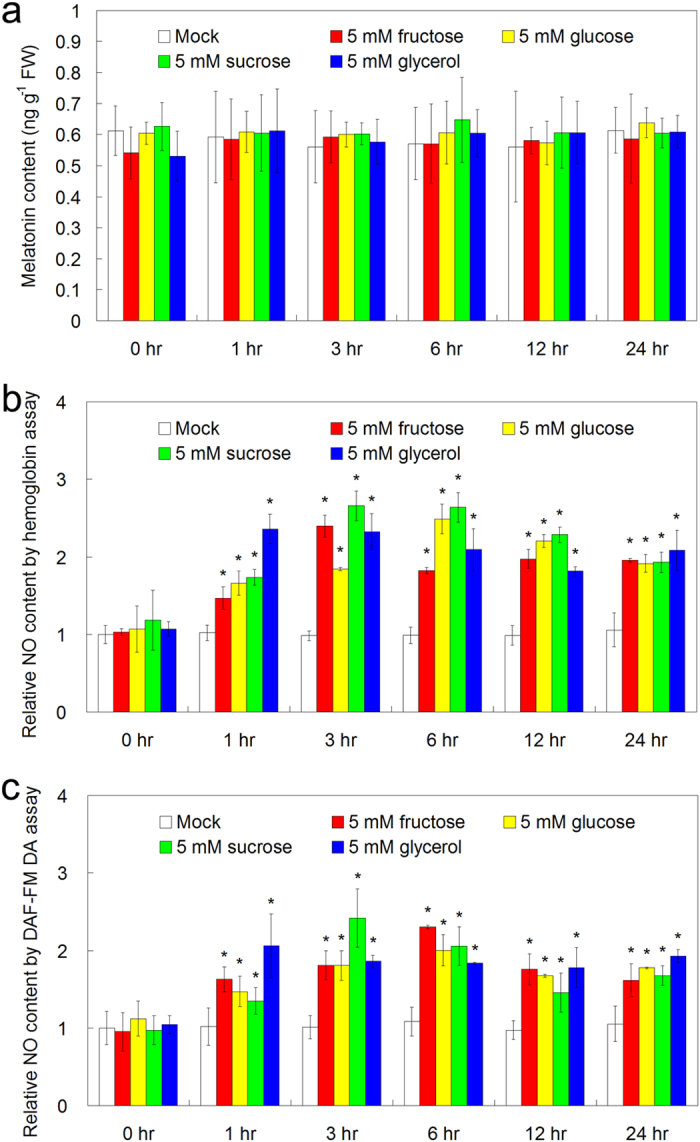
The effects of exogenous treatments of fructose, glucose, sucrose and glycerol on endogenous levels of melatonin (a) and NO (b) (c) in *Arabidopsis*. For the assay, 26-day-old soil-grown WT (Col-0) *Arabidopsis* plants were watered with nutrient solution containing mock, 5 mM fructose, 5 mM glucose, 5 mM sucrose and 5 mM glycerol from below in pots with plants for 0, 3, 6, 12 and 24 hrs, respectively, and plant leaves were used for the determination. Endogenous NO level in the leaves were assayed using the hemoglobin assay (**b**) and the DAF-FM DA assay (**c**). The results shown are the means ± SDs (n = 3). The sterisk (*) indicates significant difference in comparison to 0 hr of treatment at *p* < 0.05.

Moreover, we also examined the effects of exogenous treatments of fructose, glucose, sucrose and glycerol on endogenous levels of melatonin and NO in *Arabidopsis*. After the treatments using these sugars and sugar alcohols for 3, 6, 12 and 24 hrs, no significant difference of endogenous melatonin level was determined between mock and sugars or glycerol-treated *Arabidopsis* leaves (Fig. 7a). Using both the hemoglobin assay and the NO-sensitive fluorescence assay to detect endogenous NO, we found that 5 mM fructose, or 5 mM glucose, or 5 mM sucrose, or 5 mM glycerol treatment significantly increased endogenous NO level after 3 to 24 hrs of treatment (Fig. 7b,c).

## Discussion

Plants are exposed to changeable and complex environmental conditions, however, as sessible organisms, plants can not change their location to escape the harsh circumstances^39^,^50^,^51^,^52^,^53^,^54^,^55^,^56^,^57^. Bacterial pathogen infection is one of the most major and severe harsh stresses that influence plant growth and result in yield loss in agriculture^20^,^32^,^33^,^51^,^52^,^58^. Once a bacterial pathogen is applied, the pathogen enters the host plant tissues (usually leaves) through natural opening stomata or wounds, leading to high population levels in intercellular spaces of host plants; these results in water-soaked patches and necrotic in infected leaves^58^,^59^,^60^. In the long evolution during plant-pathogen interaction, plants have developed a variety of mechanisms to perceive, recognize and counteract pathogen entry, including pathogen-associated molecular pattern (PAMP)-triggered immunity (PTI) and effector-triggered immunity (ETI)^58^,^59^,^60^.

To date, there are three major methods to improve plant disease resistance. First is to screen and identify disease-resistant varieties from numerous wild genotypes and apply them in agriculture, and second is to investigate some major genes that confer improved disease resistance and modulate the expression of these genes by genetic breeding^51^,^52^,^61^. Moreover, screening protective molecules such as melatonin, SA, NO, hydrogen sulfide (H_2_S) in plant innate immunity and exogenous applications of them can also enhance plant disease resistance^20^,^32^,^33^,^60^,^61^,^62^,^63^,^64^,^65^. In recent years, the safety of transgenic crops has attracted many critics and many people can not accept such products^66^,^67^,^68^. Thus, exogenous applications of the protective molecules such as melatonin may be acceptable and effective.

SA signaling is the most important pathway of plant defense response against pathogen infection. These include the induction of endogenous SA in response to pathogen infection, the perception of SA by SA receptors (Nonexpressor of Pathogenesis Related Protein 3 (NPR3) and NPR4), the release of NPR1 monomers and translocation in nucleus as well as the interaction of nuclear NPR1 and TGACG sequence-specific binding protein (TGA) transcription factors, and the activation of *PRs*^58^,^59^,^60^,^69^,^70^. NO is also an important gaseous molecule and secondary messenger in plant disease response against pathogen infection^71^,^72^,^73^,^74^,^75^,^76^,^77^,^78^,^79^,^80^. On one hand, NO promotes the nuclear translocation of NPR1 and S-nitrosylation (SNO) of NPR1 and TGA1 by NO enhances the DNA binding activity of TGA1 to the promoters of *PRs*, resulting in the activation of *PRs*^51^,^61^,^71^,^72^,^73^,^74^,^75^,^76^,^77^,^78^. By comparison, SNO of respiratory burst oxidase protein D (RBOHD) by NO blunts SA biosynthesis and decreases RBOHD activity, resulting in reduced reactive oxygen intermediates and cell death during pathogen-triggered hypersensitive response in *Arabidopsis*^79^,^80^. Based on previous studies that investigated the effects of melatonin on disease resistance in WT plants, SA-deficient NahG plants and NO-deficient mutants (*noa1* and *nia1nia2*)^32^,^33^,^41^, the cooperation between melatonin and SA as well as the relation of NO and SA have been revealed, indicating that melatonin conferred enhanced plant innate immunity against bacterial pathogen in both SA- and NO-dependent pathway in *Arabidopsis*.

As a possible secondary messenger in plant stress responses, melatonin may perceive stress signaling by extensive reprogramming of the transcriptome and the proteome^19^,^23^,^26^,^42^. Weeda *et al.* and Shi *et al.* identified 1308 and 3933 increased or reduced gene transcripts due to exogenous melatonin treatment in *Arabidopsis* and in bermudagrass, respectively^23^,^26^. Wang *et al.* and Shi *et al.* identified 309 and 76 differentially expressed proteins as a result of exogenous melatonin administration in apple and in bermudagrass, respectively^19^,^42^. Moreover, some important transcription factors that are regulated by melatonin including zinc finger of *Arabidopsis thaliana* 6 (ZAT6)^24^, auxin resistant 3 (AXR3)/indole-3-acetic acid inducible 17 (IAA17)^25^, and class A1 heat shock factors (HSFA1s)^27^ are involved in melatonin-mediated freezing stress, natural leaf senescence, and heat stress responses, respectively. However, melatonin-mediated metabolomic analysis, especially melatonin-mediated metabolic homeostasis in response to bacterial pathogen, is unknown.

In this study, although 51 primary metabolites including 16 amino acids, 12 organic acids, 18 sugars and 5 sugar alcohols were reproducibly examined in *Arabidopsis* leaves after exogenous melatonin treatment and *Pst* DC3000 infection, only 7 sugars (fructose, glucose, melibose, sucrose, maltose, galatose and tagatofuranose) and glycerol commonly increased after these treatments (Figs 1, 2, 3, Table 1 and Supplemental Table S1). Fructose, glucose and sucrose are three popular sugars with wide distributions in plants^26^,^54^, so they and glycerol were chosen for further analysis. Further studies showed that exogenous fructose, glucose, sucrose and glycerol pre-treatments enhanced innate immunity against *Pst* DC3000 infection in WT plants (Fig. 4); these sugars and glycerol pre-treatments increased endogenous NO level but had no significant effect on the endogenous melatonin level (Fig. 7). These results indicated the involvement of sugars of glycerol in melatonin-mediated basal immunity, as well as in SA and NO signaling pathways.

Consistently, Thibaud *et al.* and Tsutsui *et al.* revealed the positive regulation of glucose and sucrose in plant disease resistance in SA-dependent way^81^,^82^. Both SA and NO confer enhanced disease resistance against bacterial pathogen in *Arabidopsis*, and the cooperation between them plays important roles in plant innate immunity^41^,^69^,^70^,^71^,^72^,^73^,^74^,^75^,^76^,^77^,^78^,^79^,^80^. Because melatonin confers enhanced plant innate immunity against bacterial pathogen in both SA and NO-dependent pathway in *Arabidopsis*^41^, we therefore investigated the possible cooperation among sugars and glycerol-mediated innate immunity, SA and NO signaling pathways. Interestingly, both SA and NO were essential for fructose, glucose, sucrose and glycerol-conferred enhanced innate immunity, as evidenced by the alleviated effects of them in SA-deficient NahG plants and NO-deficient *Arabidopsis* mutants (Figs 5, 6). Notably, Zhao *et al.* [2015] found that exogenous application of melatonin mediated invertase inhibitor (C/VIF)-regulated CWI activity and enhanced sucrose metabolism, thus significantly increased the productions of sucrose, glucose and fructose (by 7 to 9 fold higher)^49^. Moreover, they found that both melatonin-mediated carbohydrate metabolism including sucrose, xylose and galactose and mediated-activated SA responsive genes are contributed to pathogen resistance^49^. Both this study and Zhao’s results highlight melatonin-mediated carbohydrate metabolism in melatonin-mediated basal immunity. Additionally, Mandal *et al.* [2012] found that glycerol increases endogenous NO level and involves in NO-mediated defense signaling in *Arabidopsis*^83^. Taken together, this study is the first comprehensive metabolomic analysis including amino acids, organic acids, sugars and sugar alcohols regarding to the protective role of melatonin in innate immunity against a bacterial pathogen in *Arabidopsis*. In the meanwhile, the relationship among sugars, glycerol, SA and NO signaling pathways were partially revealed in melatonin-mediated basal immunity.

**Figure 8:**
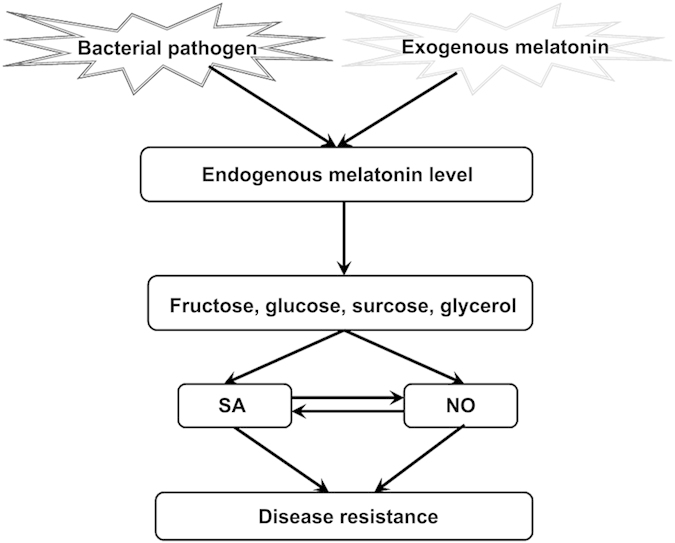
Model depicting the involvement of sugars and glycerol in melatonin-mediated innate immunity against bacterial pathogen in *Arabidopsis*.

Based on the results herein, a novel model for sugars and glycerol in melatonin*-*mediated disease resistance against bacterial pathogen in *Arabidopsis* is proposed in this study (Fig. 8). As previous described, both bacterial pathogen *Pst* DC3000 infection and exogenous application of melatonin largely increased endogenous melatonin level and accumulations of various sugars (fructose, glucose and surcose) and glycerol; these sugars and sugar alcohol confer enhanced disease resistance against *Pst* DC3000. As positive regulators of plant innate immunity, both SA and NO are essential for melatonin as well as sugars (fructose, glucose and surcose) and glycerol-mediated disease responses against *Pst* DC3000 in *Arabidopsis*.

In the current report, we identify the metabolic pathway regarding to the protective role of melatonin in innate immunity against a bacterial pathogen in *Arabidopsis*. Melatonin treatment increased the accumulations of sugars and glycerol in response to bacterial pathogen infection, and the elevated sugars and glycerol thereafter increase endogenous NO level which confer enhanced innate immunity against bacterial pathogen in SA and NO-dependent pathway in *Arabidopsis*.

## Methods

### Plant materials and growth conditions

All *Arabidopsis* seeds in the background of Columbia-0 (Col-0) ecotype were first stratificated at 4 °C for 3 days in darkness, and thereafter were sown in soil in the growth chamber. The growth chamber was controlled at 23 ± 2 °C, with the irradiance of about 120 μmol quanta m^−2^ s^−1^ and 65% relative humidity under 16 hr light and 8 hr dark cycles. Nutrient solution was watered in the soil twice every week, to keep plant growth. The NO-deficient mutants [*noa1* (CS6511) and *nia1nia2* (CS2356)] were from *Arabidopsis* Biological Resource Center (ABRC), and SA-deficient NahG (the salicylate hydroxylase-expressing transgenic plant) has been described in Gaffney *et al.*^50^.

### Infection of bacterial pathogen *Pst* DC3000

The bacterial pathogen strain of *Pst* DC3000 was used for the assay plant innate immunity as previously described^41^,^51^,^52^. The strain of *Pst* DC3000 was streaked out on King’s B (KB) medium containing 50 μg ml^−1^ rifampicin at 28 °C for 2 days, and fresh bacteria was transferred to new KB liquid culture containing 50 μg ml^−1^ rifampicin at 28 °C for 8 to 12 hrs, until bacterial culture reached OD_600_ of 0.6 to 1.0. Thereafter the virulent *Pst* DC3000 was diluted to OD_600_ of 0.002, and the bacterial suspension containg 10 mM MgCl_2_ and 0.05% silwet L-77 were infected in the abaxial side of 28-day-old plant leaves. After syringe infection, plants were covered with plastic dome to maintain humidity for 2 days at 23 °C in the growth chamber.

At 0, 2 and 4 dpi, syringe infected leaves were harvested and placed in 70% ethanol solution for 1 minute with gently mixed occasionally, thereafter the leaves were removed and rinsed in sterile distilled water for 1 minute, the leaves are then dry on paper towel. At least 15 independent leaf discs of 1 cm^2^ within the infected area in each treatment of one independent experiment were excised, and the bacterial populations in the leave discs were determined in KB medium containing 1.5% (m/v) agar and 50 μg ml^−1^ rifampicin using 10 μl five 10-fold dilutions of homogenate of *Pst* DC3000-infected leaves as described in Shi *et al.*^41^,^51^,^52^. Six biological repeats were performed for the innate disease resistance assay.

### Extraction, identification and quantification of primary metabolites in *Arabidopsis* leaves

Extraction, identification and quantification of primary metabolites of plant leaves were performed as previously described^53^,^54^. Briefly, 100 mg plant leaves was ground in liquid nitrogen and the dry powder was transferred to a new EP tube, and 1.4 ml pre-cooled 100% methanol was added and vortexed for 10 seconds, and thereafter 60 μl 0.2 mg mL^−1^ ribitol was added as an internal standard and vortexed for 10 another 10 seconds. After shaking at 70 °C for 10 min, the mixture was centrifuged at 12560 g for 10 min, and the supernatant was transferred to a new tube with 750 μl chloroform and 1.4 ml dd H_2_O. After vortexed for 30 seconds, the mixture was centrifuged at 12560 for 10 min, and 150 μl supernatant was transferred to a new EP tube with 40 μl methoxyamination reagent. The mixture was shaked at 37 °C for 2 hrs and 70 μl MSTFA reagent was added for shaking at 37 °C for another 0.5 hr.

For each sample in every biological repeat, 1 μl derivatizated extract was injected into a DB-5MS capillary (30 m × 0.25 mm × 0.25 μm, Agilent J&W GC column, California, USA). The GC-TOF-MS analysis was performed as previously described^54^, which was determined using electron impact ionization (70 eV) in full scan mode (*m/z* from 30 to 550). Different metabolites were identified by comparing retention time index specific masses from samples with reference spectra in mass spectral libraries (NIST 2005, Wiley 7.0), and quantified based on the pre-added internal standard (ribitol).

### Hierarchical cluster analysis and construction of heatmap

The hierarchical cluster analysis of different primary metabolites in *Arabidopsis* leaves with various treatments was performed using CLUSTER program (http://bonsai.ims.u-tokyo.ac.jp/~mdehoon/software/cluster/), and then the heatmap was constructed using Java Treeview (http://jtreeview.sourceforge.net/).

### Quantification of endogenous melatonin and NO levels in *Arabidopsis* leaves

Extraction and quantification of endogenous melatonin in *Arabidopsis* leaves were determined using melatonin enzyme-linked immunosorbent assay (ELISA) Kit (EK-DSM; Buhlmann Laboratories AG, Schonenbuch, Switzerland) as previously described^6^,^24^,^25^,^26^,^27^,^41^,^42^. Extraction and quantification of endogenous NO in *Arabidopsis* leaves were determined using the hemoglobin assay by examining the conversion of oxyhemoglobin to methemoglobin spectrophotometrically and the NO-sensitive fluorescence assay using 3-Amino,4-aminomethyl-2′,7′-difluorescein, diacetate (DAF-FM DA) as previously described^41^,^51^,^55^,^56^,^57^.

### Statistical analysis

All the experiments were repeated at least three biological repeats with similar results, and plant leave samples in each biological repeat were extracted from at least 10 plants. All the data were expressed as means ± SDs of the biological repeats. The statistical analysis was performed using AVOVA and student t-test, and *p* < 0.05 was considered as significant difference that was marked with asterisk (*).

## Additional Information

**How to cite this article**: Qian, Y. *et al.* Comparative metabolomic analysis highlights the involvement of sugars and glycerol in melatonin-mediated innate immunity against bacterial pathogen in *Arabidopsis*. *Sci. Rep.* **5**, 15815; doi: 10.1038/srep15815 (2015).

## Supplementary Material

Supplementary Dataset

## Figures and Tables

**Table 1:** Concentrations of 18 sugars and 5 sugar alcohols in response to melatonin and *Pst* DC3000 treatments in *Arabidopsis*. The concentrations of metabolites were expressed as μg g^−1^ FW. The data represent the means of three biological repeats ± SDs. Gray background indicates significant increased metabolite in comparison to 0 hr.

No.	Sugars or sugar alcohols	0 hr	Melatonin-6 hr	Melatonin-12 hr	Melatonin-24 hr	*Pst* DC3000-6 hr	*Pst*DC3000-12 hr
1	Allose	3.53 ± 0.54	3.60 ± 0.62	7.84 ± 0.59	5.39 ± 0.86	3.51 ± 0.11	3.79 ± 0.13
2	Arabinose	8.62 ± 2.16	8.22 ± 1.30	8.17 ± 0.69	7.04 ± 0.11	7.24 ± 0.44	8.79 ± 0.68
3	Cellobiose	9.34 ± 1.26	7.13 ± 0.68	8.91 ± 0.51	8.32 ± 0.43	8.45 ± 0.58	10.12 ± 0.89
4	Fructose	74.30 ± 7.20	112.38 ± 11.48	158.10 ± 27.24	131.08 ± 3.16	134.27 ± 7.11	268.58 ± 35.93
5	Galatose	4.24 ± 0.28	4.38 ± 0.88	4.88 ± 1.47	31.13 ± 2.67	26.29 ± 1.30	17.96 ± 3.29
6	Gentiobiose	12.09 ± 0.88	12.35 ± 0.34	13.70 ± 0.96	11.41 ± 0.19	12.56 ± 0.53	12.91 ± 0.52
7	Glucose	8.61 ± 1.47	18.31 ± 2.30	22.02 ± 5.79	11.40 ± 0.53	7.84 ± 1.44	20.18 ± 1.55
8	Lactose	6.19 ± 0.61	5.66 ± 1.69	7.15 ± 0.92	6.89 ± 0.66	8.63 ± 1.92	7.83 ± 0.47
9	Lactulose	22.20 ± 2.15	22.33 ± 2.70	23.07 ± 2.34	21.78 ± 2.08	20.14 ± 2.10	25.08 ± 4.20
10	Maltose	6.55 ± 2.31	6.67 ± 2.47	5.70 ± 1.99	18.91 ± 2.19	20.28 ± 1.16	20.89 ± 3.16
11	Mannobiose	16.79 ± 0.91	17.22 ± 0.94	18.20 ± 1.22	15.45 ± 0.47	17.57 ± 2.07	18.54 ± 0.64
12	Mannose	58.09 ± 4.50	92.19 ± 13.16	155.53 ± 24.37	46.63 ±9.43	48.21 ± 9.27	52.71 ± 2.65
13	Melibose	25.66 ± 3.15	25.70 ± 2.59	41.60 ± 3.74	34.10 ± 1.58	38.43 ± 1.80	38.84 ± 1.64
14	Sucrose	32.15 ± 2.76	32.91 ± 3.08	55.95 ± 4.17	70.88 ± 7.18	37.25 ± 2.81	95.68 ± 9.59
15	Tagatofuranose	60.10 ± 9.91	41.54 ± 17.84	54.06 ± 5.12	95.59 ± 12.68	82.05 ± 11.72	80.99 ± 1.88
16	Talose	9.70 ± 3.76	15.14 ± 7.53	8.18 ± 1.51	6.47 ± 2.58	12.53 ± 9.07	13.34 ± 7.23
17	Trehalose	3.45 ± 0.74	3.31 ± 1.14	2.85 ± 0.13	2.39 ± 0.05	6.77 ± 0.21	4.71 ± 0.18
18	Turanose	4.07 ± 0.46	3.88 ± 1.27	8.46 ± 2.34	5.75 ± 0.23	9.10 ± 1.46	6.04 ± 0.29
19	Dulcitol	6.07 ± 1.42	6.15 ± 1.21	6.12 ± 1.34	4.38 ± 0.61	5.87 ± 1.16	6.01 ± 1.51
20	Galactinol	4.92 ± 1.50	9.08 ± 3.72	15.40 ±1.11	15.08 ± 0.17	7.24 ± 1.45	6.98 ± 2.22
21	Glycerol	17.29 ± 1.29	24.23 ± 2.92	29.32 ± 1.99	68.52 ± 2.48	54.79 ± 1.35	55.58 ± 3.36
22	Myo-Inositol	39.65 ± 5.75	33.45 ± 0.38	48.76 ± 17.61	28.43 ± 0.69	35.71 ± 10.96	28.52 ± 4.46
23	Sorbitol	22.83 ± 2.44	21.53 ± 2.69	25.94 ± 4.31	22.06 ± 3.71	21.39 ± 3.21	25.09 ± 3.06
