# Supplementary material for: Comparative metabolomic analysis highlights the involvement of sugars and glycerol in melatonin-mediated innate immunity against bacterial pathogen in Arabidopsis
Source: Sci Rep. 2015 Oct 28;5:15815. doi: 10.1038/srep15815 (PMC4623600; doi:10.1038/srep15815)
Supplement: Supplementary Dataset [file srep15815-s1.doc]

**Comparative metabolomic analysis highlights the involvement of sugars and glycerol in melatonin-mediated innate immunity against bacterial pathogen in *Arabidopsis***

**Yongqiang Qian**^1, 2^**, Dun-Xian Tan**^3^**, Russel J. Reiter**^3^**, Haitao Shi**^1, *^

^1^ Hainan Key Laboratory for Sustainable Utilization of Tropical Bioresources, College of Agriculture, Hainan University, Haikou, 570228, China,

^2^ State Key Laboratory of Tree Genetics and Breeding, Research Institute of Forestry, Chinese Academy of Forestry, Beijing, 100091, China,

^3^ Department of Cellular and Structural Biology, The University of Texas Health Science Center, San Antonio, TX, USA.

**Supplementary Table S1. Concentrations of 16 amino acids and 12 organic acids in response to melatonin and *Pst* DC3000 treatments in *Arabidopsis*.** The concentrations of metabolites were expressed as µg g^-1^ FW. The data represent the means of three biological repeats ± SDs. Gray background indicates significant increased metabolite in comparison to 0 hr.

| No. | Amino acids or organic acids | 0 hr | Melatonin-6 hr | Melatonin-12 hr | Melatonin-24 hr | *Pst* DC3000-6 hr | *Pst* DC3000-12 hr |
| --- | --- | --- | --- | --- | --- | --- | --- |
| 1 | Alanine | 27.31 ± 5.56 | 21.23 ± 3.14 | 29.50 ± 4.29 | 25.91 ± 1.39 | 28.18 ± 2.48 | 28.33 ± 3.41 |
| 2 | Asparagine | 19.29 ± 1.25 | 17.08 ± 1.74 | 19.74 ± 2.42 | 18.48 ± 2.44 | 20.09 ± 1.01 | 22.94 ± 2.64 |
| 3 | Aspartic acid | 10.61 ± 1.02 | 10.33 ± 2.05 | 13.09 ± 1.74 | 12.51 ± 1.40 | 13.14 ± 1.30 | 9.47 ± 1.42 |
| 4 | Citrulline | 24.17 ± 2.90 | 26.48 ± 2.42 | 72.02 ± 51.51 | 19.43 ± 2.91 | 22.19 ± 2.36 | 29.44 ± 5.07 |
| 5 | Glutamic acid | 23.68 ± 1.48 | 23.58 ± 3.77 | 30.50 ± 6.42 | 22.97 ± 1.32 | 35.13 ± 8.85 | 32.89 ± 5.40 |
| 6 | Glutamine | 39.01 ± 7.88 | 30.60 ± 3.01 | 35.85 ± 2.56 | 36.42 ± 3.86 | 34.11 ± 6.43 | 42.34 ± 5.64 |
| 7 | Glycine | 6.53 ± 0.44 | 5.90 ± 0.60 | 5.59 ± 0.82 | 5.92 ± 0.76 | 5.25 ± 2.63 | 7.14 ± 1.00 |
| 8 | Isoleucine | 30.83 ± 1.56 | 30.47 ± 1.96 | 32.28 ± 1.71 | 29.84 ± 1.58 | 55.42 ± 4.66 | 33.17 ± 4.22 |
| 9 | Leucine | 1.84 ± 0.23 | 1.84 ± 0.08 | 2.07 ± 0.22 | 1.83 ± 0.39 | 2.06 ± 0.27 | 2.12 ± 0.22 |
| 10 | Lysine | 37.08 ± 2.43 | 38.26 ± 4.98 | 38.39 ± 10.37 | 31.25 ± 7.05 | 35.84 ± 7.44 | 37.58 ± 4.17 |
| 11 | Norvaline | 3.35 ± 0.60 | 3.03 ± 0.44 | 3.64 ± 0.27 | 1.55 ± 0.10 | 3.30 ± 0.28 | 2.91 ± 0.32 |
| 12 | Ornithine | 8.30 ± 0.88 | 6.37 ± 0.69 | 7.48 ± 1.05 | 8.25 ± 2.18 | 9.20 ± 2.01 | 8.31 ± 1.68 |
| 13 | Proline | 38.06 ± 8.17 | 31.11 ± 4.10 | 86.16 ± 16.83 | 70.35 ± 7.07 | 29.39 ± 6.34 | 47.76 ± 11.41 |
| 14 | Serine | 24.87 ± 3.63 | 22.45 ± 1.06 | 23.20 ± 2.06 | 21.69 ± 2.60 | 25.37 ± 4.22 | 30.27 ± 3.13 |
| 15 | Threonine | 18.83 ± 3.19 | 18.55 ± 2.69 | 22.57 ± 1.47 | 19.28 ± 2.60 | 18.91 ± 0.76 | 19.51 ± 1.96 |
| 16 | Valine | 3.73 ± 0.25 | 2.99 ± 0.58 | 4.21 ± 0.51 | 3.63 ± 0.87 | 4.17 ± 0.74 | 4.22 ± 0.15 |
| 17 | Acetic acid | 29.72 ± 5.58 | 20.98 ± 10.13 | 29.42 ± 2.70 | 23.69 ± 3.16 | 29.52 ± 1.33 | 40.82 ± 4.88 |
| 18 | Ascorbic acid | 10.28 ± 1.07 | 8.73 ± 1.27 | 8.59 ± 1.19 | 8.23 ± 1.91 | 8.16 ± 1.21 | 9.51 ± 0.93 |
| 19 | Butanoic acid | 2.78 ± 0.23 | 2.70 ± 0.12 | 3.27 ± 0.47 | 2.32 ± 0.23 | 2.62 ± 0.42 | 3.21 ± 0.40 |
| 20 | Cinnamic acid | 28.64 ± 4.92 | 28.46 ± 4.00 | 29.98 ± 2.91 | 22.92 ± 2.27 | 27.38 ± 3.14 | 28.94 ± 2.44 |
| 21 | Ethanedioic acid | 3.17 ± 0.32 | 2.90 ± 0.22 | 3.28 ± 0.31 | 2.75 ± 0.38 | 3.16 ± 0.22 | 3.39 ± 0.33 |
| 22 | Gluconic acid | 6.85 ± 0.95 | 5.81 ± 0.62 | 6.99 ± 1.58 | 7.00 ± 0.48 | 7.10 ± 0.94 | 7.67 ± 1.63 |
| 23 | Hexadecanoic acid | 32.01 ± 3.73 | 28.23 ± 6.17 | 28.47 ± 8.70 | 22.79 ± 4.23 | 28.35 ± 3.29 | 30.04 ± 8.21 |
| 24 | Octadecanoic acid | 5.77 ± 0.78 | 4.93 ± 0.70 | 5.10 ± 1.19 | 4.48 ± 0.97 | 5.80 ± 1.52 | 4.87 ± 1.27 |
| 25 | Pentanedioic acid | 14.07 ± 2.26 | 8.89 ± 1.14 | 11.94 ± 2.27 | 12.27 ± 2.18 | 10.59 ± 1.00 | 10.17 ± 2.97 |
| 26 | Phosphoric acid | 14.91 ± 2.89 | 13.75 ± 2.09 | 15.81 ± 2.37 | 11.76 ± 4.26 | 12.13 ± 2.46 | 17.11 ± 0.56 |
| 27 | Propanoic acid | 5.96 ± 0.96 | 4.38 ± 0.79 | 5.21 ± 1.14 | 5.87 ± 0.65 | 7.55 ± 0.59 | 7.05 ± 1.28 |
| 28 | Threonic acid | 25.63 ± 0.61 | 24.17 ± 2.52 | 27.89 ± 5.31 | 24.39 ± 2.75 | 26.20 ± 5.00 | 24.19 ± 4.39 |
